# Domain enhanced lookup time accelerated BLAST

**DOI:** 10.1186/1745-6150-7-12

**Published:** 2012-04-17

**Authors:** Grzegorz M Boratyn, Alejandro A Schäffer, Richa Agarwala, Stephen F Altschul, David J Lipman, Thomas L Madden

**Affiliations:** 1 National Center for Biotechnology Information, National Library of Medicine, National Institutes of Health, Building 38A, 8600 Rockville Pike, Bethesda, MD, 20894, USA

## Abstract

**Background:**

BLAST is a commonly-used software package for comparing a query sequence to a database of known sequences; in this study, we focus on protein sequences. Position-specific-iterated BLAST (PSI-BLAST) iteratively searches a protein sequence database, using the matches in round *i* to construct a position-specific score matrix (PSSM) for searching the database in round *i* + 1. Biegert and Söding developed Context-sensitive BLAST (CS-BLAST), which combines information from searching the sequence database with information derived from a library of short protein profiles to achieve better homology detection than PSI-BLAST, which builds its PSSMs from scratch.

**Results:**

We describe a new method, called domain enhanced lookup time accelerated BLAST (DELTA-BLAST), which searches a database of pre-constructed PSSMs before searching a protein-sequence database, to yield better homology detection. For its PSSMs, DELTA-BLAST employs a subset of NCBI’s Conserved Domain Database (CDD). On a test set derived from ASTRAL, with one round of searching, DELTA-BLAST achieves a ROC_5000_ of 0.270 vs. 0.116 for CS-BLAST. The performance advantage diminishes in iterated searches, but DELTA-BLAST continues to achieve better ROC scores than CS-BLAST.

**Conclusions:**

DELTA-BLAST is a useful program for the detection of remote protein homologs. It is available under the “Protein BLAST” link at http://blast.ncbi.nlm.nih.gov.

**Reviewers:**

This article was reviewed by Arcady Mushegian, Nick V. Grishin, and Frank Eisenhaber.

## Background

Popular sequence alignment algorithms, such as BLAST [1] or FASTA [2], use substitution score matrices to measure similarity between two amino acid or nucleotide sequences. In a 20 × 20 protein substitution matrix, each element *s*_*ij*_ is a score derived from the probability that, in homologous sequences, amino acids *i* and *j* descend from a common ancestor. Sequence similarity searches generally perform better at detecting distantly related homologs when they use either matrices specialized for particular protein classes [3-11], or position-specific score matrices (PSSMs) [12-23].

A PSSM associated with a sequence of length *l* is an *l* × 20 matrix, where element *s*_*ij*_ is derived from the probability that related sequences have amino acid *j* at PSSM position *i*. A PSSM is constructed from a multiple sequence alignment (MSA) of related proteins, and models the amino acid substitutions particular to a specific protein family and sequence position.

Separate multiple alignment programs may be used to construct the MSAs from which PSSMs are derived [18]. Position Specific Iterated BLAST (PSI-BLAST) [23] introduced the strategy of automatically generating MSAs and their associated PSSMs from the results of database searches, in an iterative manner. The output of iteration *i* is used to construct a PSSM, and search the sequence database in iteration *i* + 1. Biegert and Söding [24] developed Context-Specific BLAST (CS-BLAST), which computes an initial PSSM using a query sequence and a library of short profiles. To construct this library, the authors first construct a large number of MSAs by aligning subsets of sequences from the whole non-redundant protein database (NR) [25] with one another, using two iterations of PSI-BLAST. These MSAs, converted into amino acid frequency profiles, are divided into short windows and clustered to create the profile library. CS-BLAST achieves better sensitivity than PSI-BLAST.

One can also use an existing collection of pre-constructed MSAs to derive a PSSM. We take a related approach here, using the Conserved Domain Database (CDD) [26], an NCBI resource for identifying conserved domains within protein sequences. This database includes manually curated domain models that are refined using protein 3D structures, as well as models constructed from clusters of related sequences with unknown structure. Each conserved domain (CD), represented by an MSA of homologous sequence segments, is converted to a PSSM to facilitate efficient search [26]. Software tools for searching collections of PSSMs include HMMER [27], IMPALA [28], RPS-BLAST, and GLOBAL [29].

**Figure 1:**
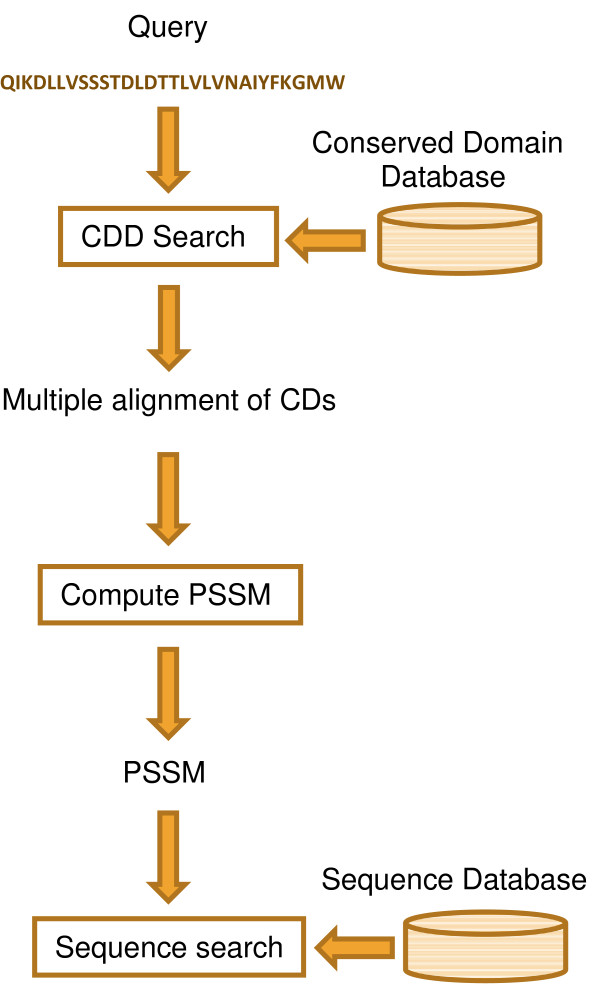
** Overview of sequence search with DELTA-BLAST.** DELTA-BLAST searches CDD with the supplied query, uses aligned domains to compute a PSSM and searches a sequence database with this PSSM.

We describe Domain Enhanced Look-up Time Accelerated BLAST (DELTA-BLAST), a new tool that first uses RPS-BLAST to align a query sequence to conserved domains in CDD, and then performs a sequence database search using a PSSM derived from the aligned domains. The PSSM construction method is similar to that of PSI-BLAST, but begins by aligning the query to CDs rather than to individual sequences. Figure 1 shows an overview of DELTA-BLAST’s strategy.

Our primary goals for DELTA-BLAST are to make use of a PSSM in the search (as in PSI-BLAST) to find more homologs, but to avoid the time spent in the initial BLASTP search. DELTA-BLAST also allows us to explore whether it is better to use longer homologous alignments to quickly construct a PSSM than the short profiles of Biegert and Söding [24]. In future work, it may serve as a platform to experiment with different methods for quickly finding initial matches to a query that can then be used to construct a PSSM.

We demonstrate that, when used with CDD, DELTA-BLAST is more sensitive than both CS-BLAST and PSI-BLAST. This result speaks not just to DELTA-BLAST’s effectiveness, but also to the extensiveness of the CDD collection.

DELTA-BLAST is fully integrated with the NCBI BLAST website and the stand-alone BLAST+ package. It is available from the “Protein BLAST” link at the NCBI BLAST website (http://blast.ncbi.nlm.nih.gov). A DELTA-BLAST search on the website can be followed up by PSI-BLAST iterations or the results can be processed further by the distance tree or multiple alignment tools. A new program named *deltablast* will be is part of the command-line BLAST+ package starting with the 2.2.26+ release. Source code and applications for popular platforms are available at ftp://ftp.ncbi.nlm.nih.gov/blast/executables/blast+/LATEST/.

## Results

This section compares the performance of BLASTP, CS-BLAST, PSI-BLAST, and DELTA-BLAST. We assessed the homology-detection effectiveness of these methods using search results for the ASTRAL Compendium for Sequence and Structure Analysis [30] and the Structural Classification of Proteins (SCOP) [31] databases. A database of 10,569 sequences was searched using a set of 4,852 queries. To assess not only search sensitivity but also the quality of the alignments produced, we compared program-generated alignments of 10,006 pairs of 3D domains from the superfamily subset of the SABmark set [32] to these pairs’ reference alignments. Finally, to further assess algorithm sensitivity, we analyzed the numbers of true positive results yielded by the search methods, articulated further by their CDD annotation.

### Homology detection

We evaluated the homology-detection performance of DELTA-BLAST, BLASTP, and CS-BLAST using a popular measure of retrieval accuracy, the Receiver Operating Characteristic (ROC) [33]. Specifically, we employed the ROC_*n*_ score, calculated by pooling the alignments generated by all queries, ordered by *E*-value, and then considering results only up to the *n*th false positive [34].

**Figure 2:**
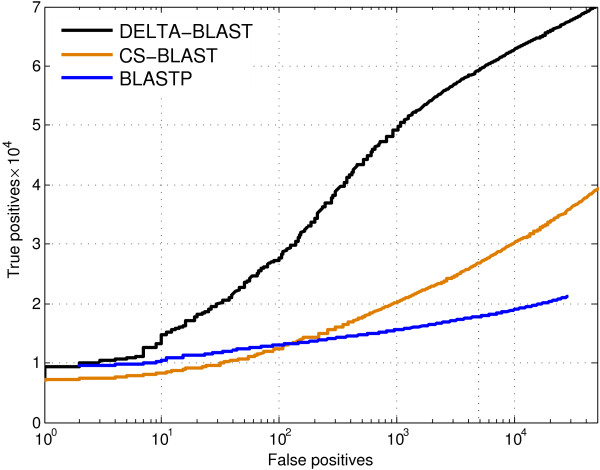
** Number of true positives vs. number of false positives for DELTA-BLAST, CS-BLAST and BLASTP.** The searched database was created using ASTRAL 40 sequences for SCOP version 1.75. To create the query set, we sorted the SCOP domains in lexicographic order and selected even numbered sequences for the test query set. We excluded from the query set any sequence that was the sole member of its superfamily in ASTRAL 40. We considered a query and database sequence to be homologs if they belonged to the same superfamily, and non-homologs if they belonged to different folds. The search results generated by all queries were pooled and ordered by E-value. The database and the query set consisted of 10,569 and 4852 sequences, respectively.

The ROC_5000_ and ROC_10,000_ scores for DELTA-BLAST (Table 1) are 2.2 times higher than those for CS-BLAST, and 3.2 times higher than those for BLASTP. For 5000 false positives (approximately one per query), DELTA-BLAST finds about twice as many homologs as CS-BLAST and three times as many as BLASTP (Figure 2).

**Table 1:** Retrieval accuracy for BLASTP, CS-BLAST, and DELTA-BLAST

**Method**	**ROC**_**5000**_		**ROC**_**10,000**_	
BLASTP	0.084	(±0.0001)	0.089	(±0.0001)
CS-BLAST	0.116	(±0.0004)	0.131	(±0.0003)
DELTA-BLAST	0.270	(±0.0007)	0.291	(±0.0005)

**Figure 3:**
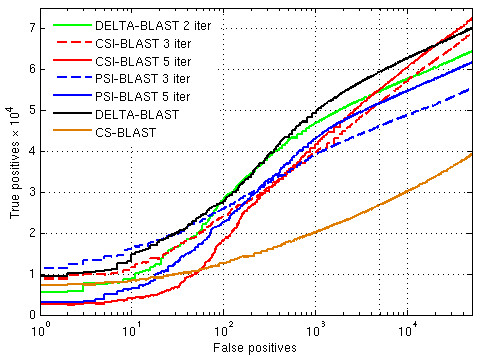
** Number of true positives vs. number of false positives for PSI-BLAST, iterated DELTA-BLAST, CSI-BLAST, DELTA-BLAST, and CS-BLAST.** See the legend of Figure 2.

We also compared iterated search methods: PSI-BLAST, Context-Specific Iterated BLAST (CSI-BLAST) [24], and iterated DELTA-BLAST (see **Methods**). Table 2 and Figure 3 summarize the results. For DELTA-BLAST, 3, 4, and 5 iterations yield results no better then 2, but the program still outperforms CS-BLAST and PSI-BLAST. For corresponding numbers of iterations, CSI-BLAST outperforms PSI-BLAST.

**Table 2:** Retrieval accuracy for PSI-BLAST, CSI-BLAST, and iterated DELTA-BLAST

**Method**	**ROC**_**5000**_		**ROC**_**10,000**_	
PSI-BLAST 2 iter	0.175	(±0.0004)	0.187	(±0.0003)
PSI-BLAST 3 iter	0.212	(±0.0005)	0.227	(±0.0003)
PSI-BLAST 4 iter	0.228	(±0.0006)	0.245	(±0.0004)
PSI-BLAST 5 iter	0.234	(±0.0007)	0.253	(±0.0004)
CSI-BLAST 2 iter	0.197	(±0.0007)	0.221	(±0.0005)
CSI-BLAST 3 iter	0.225	(±0.0008)	0.252	(±0.0005)
CSI-BLAST 4 iter	0.233	(±0.0009)	0.262	(±0.0006)
CSI-BLAST 5 iter	0.237	(±0.0009)	0.266	(±0.0006)
DELTA-BLAST 2 iter	0.251	(±0.0006)	0.269	(±0.0004)

Alignment methods may show different behaviors for different protein types. Therefore, we divided the test set by SCOP class and computed ROC_*n*_ score for the pooled search results for each class, with *n* equal to the number of queries ineach subset. DELTA-BLAST yields the largest ROC_*n*_ scores for all SCOP classes, except for small proteins (Table 3).

**Table 3:** Retrieval accuracy for DELTA-BLAST, BLASTP, CS-BLAST, and 5 iterations of PSI-BLAST and CSI-BLAST, across SCOP classes

**Class**	**BLASTP**		**CS-BLAST**		**DELTA-BLAST**		**PSI-BLAST**		**CSI-BLAST**	
A	0.061	(0.0003)	0.084	(0.0005)	**0.192**	(0.0009)	0.172	(0.0008)	**0.192**	(0.0012)
B	0.095	(0.0003)	0.108	(0.0004)	**0.356**	(0.0013)	0.285	(0.0022)	0.267	(0.0028)
C	0.062	(0.0002)	0.096	(0.0009)	**0.189**	(0.0015)	0.163	(0.0013)	0.173	(0.0015)
D	0.166	(0.0004)	0.198	(0.0007)	**0.471**	(0.0009)	0.443	(0.0009)	0.452	(0.0008)
E	0.263	(0.0013)	0.276	(0.0011)	**0.459**	(0.0046)	0.415	(0.0023)	0.439	(0.0029)
F	0.376	(0.0026)	0.391	(0.0034)	**0.563**	(0.0029)	0.474	(0.0019)	0.530	(0.0072)
G	0.066	(0.0010)	0.059	(0.0014)	0.120	(0.0010)	**0.133**	(0.0010)	0.123	(0.0021)

We divided the pooled search results into subsets, according to the SCOP protein class of the query and computed ROC_*n*_ scores for each subset, with *n* equal to the number of queries in each class. See the legend of Figure 2 for data set description. The rows represent SCOP protein classes: A - All alpha proteins (866 queries), B - All beta proteins (1034 queries), C - Alpha and beta proteins a/b (1310 queries), D - Alpha and beta proteins a + b (1219 queries), E - Multi-domain proteins (78 queries), F - Membrane and cell surface proteins and peptides (64 queries), and G - Small proteins (284 queries). The columns show ROC_*n*_ scores and standard errors for each method. The largest scores per row are in bold font.

**Figure 4:**
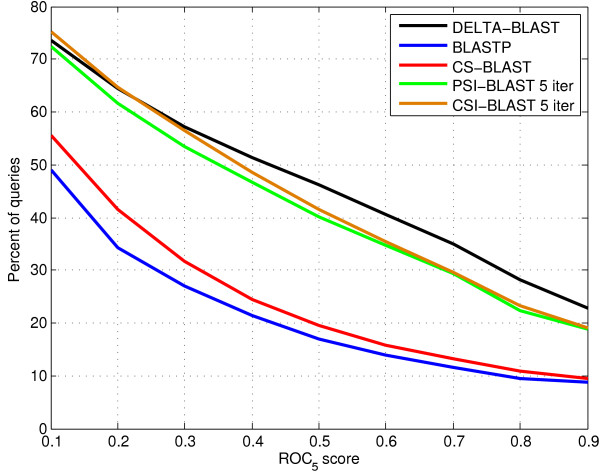
** Percentage of queries exceeding a ROC**_**5**_**score vs. that score for DELTA-BLAST, BLASTP, CS-BLAST, PSI-BLAST, and CSI-BLAST.** We computed a separate ROC_5_ score for the search results of each query and counted the number of queries that yield a ROC_5_ score above 0.1, 0.2, …, 0.9. See the legend of Figure 2 for data set description.

We also compared ROC_5_ scores for search results of each individual query. Figure 4 presents the percentage of queries exceeding a ROC_5_ score vs. that score. A ROC_5_ score close to zero implies higher ranks for false positives than true positives among the top search results. A ROC_5_ of 0.5 denotes mixed ranks, and a score close to one represents results with most true positives followed by false positives.

DELTA-BLAST search results yield ROC_5_ scores exceeding values between 0.3 and 0.9 for the largest number of queries. The DELTA-BLAST improvement over PSI-BLAST and CSI-BLAST is the largest for the large scores (between 0.6 and 0.9). The improvement of CS-BLAST over BLASTP is the largest for low ROC_5_ scores (between 0.1 and 0.2). DELTA-BLAST yields search results with ROC_5_ scores above 0.9 for about 23% of the test queries, while PSI-BLAST and CSI-BLAST do so for less than 20%, and CS-BLAST and BLASTP for below 10%.

### Alignment quality

We assessed the quality of the alignments produced by DELTA-BLAST, BLASTP, and CS-BLAST by comparing these alignments to reference structure alignments, using two standard metrics – sensitivity and precision. Briefly, sensitivity measures the fraction of the structural alignment that is correctly recovered by a given sequence alignment, while precision measures the fraction of a sequence alignment that correctly reproduces the structural alignment. More details are given in the **Methods** section.

**Figure 5:**
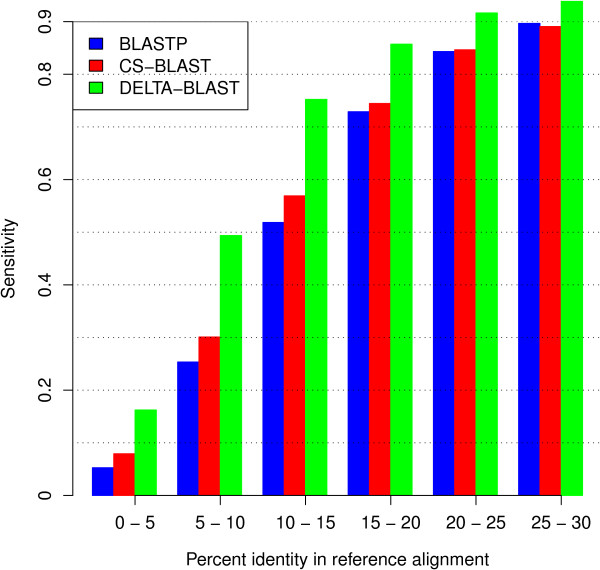
** Alignment sensitivity of BLASTP, CS-BLAST, and DELTA-BLAST.** Sensitivity measures the fraction of a reference alignment correctly recovered by a sequence alignment. Sequences and their reference alignments from the SABmark superfamily set were used to measure sensitivity. We used only reference alignments with sequence identity below 30% between sequences that did not correspond to SCOP domains present in the training set used to tune DELTA-BLAST parameters. Additionally, we removed reference alignments with fewer than five aligned pairs of residues. The data set contained 10,006 alignments between 2,379 sequences.

**Figure 6:**
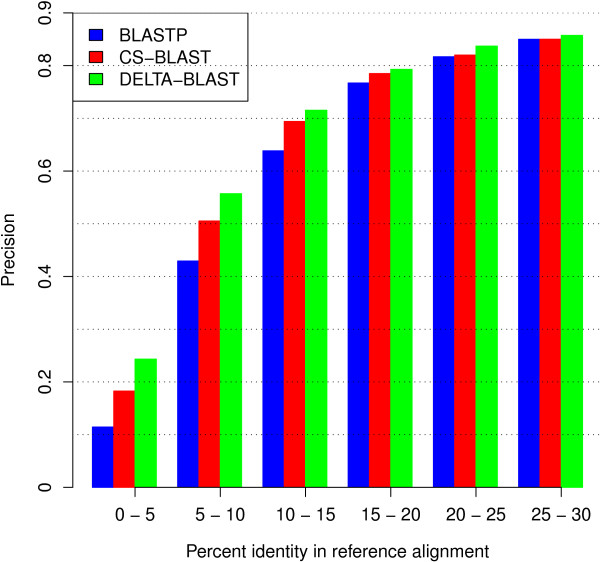
** Alignment precision of BLASTP, CS-BLAST, and DELTA-BLAST.** Precision measures the fraction of a sequence alignment that correctly reproduces a reference alignment. See the legend of Figure 5 for the data set description.

Figures 5 and 6 show the average sensitivity and precision of the programs, with results grouped by reference alignment sequence identity. On average, for the complete range of sequence identities shown in Figure 5, DELTA-BLAST produces alignments with greater sensitivity. For identities between 5% and 20% the mean sensitivity of DELTA-BLAST alignments is larger by at least 0.1 than that of the other methods. By the measure of precision (Figure 6), DELTA-BLAST also outperforms both BLASTP and CS-BLAST, although by smaller margins. The largest differences appear at low sequence identity; for identities over 20%, the mean precisions of all methods are similar.

### Comparison of nominal E-values

**Figure 7:**
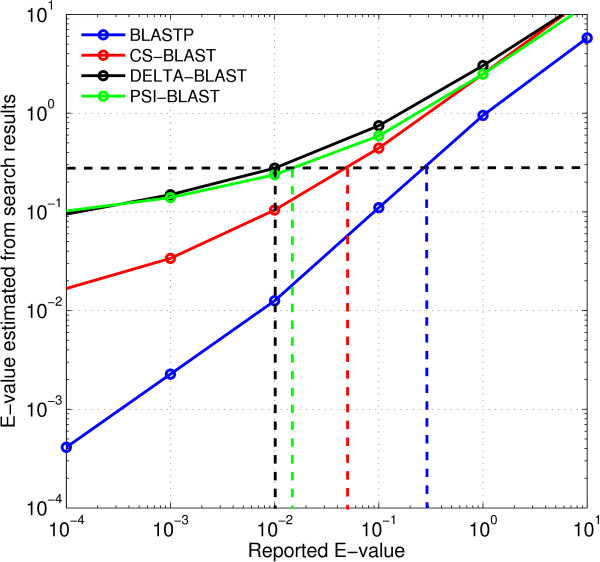
** Average number of false positives as a function of nominal E-value.** The plot shows the relationship between the nominal *E*-values reported by the search methods and actual *E*-values, estimated from search results. For a particular search method and nominal *E*-value *x*, the actual *E*-value is estimated by the mean number of false positive alignments returned with reported *E*-value ≤ *x*. The vertical dashed lines show nominal *E*-value thresholds at which the various search methods return 0.3 false positives per query (shown by the horizontal dashed line).

The *E*-value of an alignment is the expected number of chance alignments with a score at least as high. Figure 7 presents the average number of false positive results with scores exceeding nominal *E*-values for DELTA-BLAST, PSI-BLAST, BLASTP, and CS-BLAST. Ideally, the reported or nominal *E*-value should be close to the number of such false positive alignments.

BLASTP estimates *E*-values relatively accurately on this data set. CS-BLAST underestimates *E*-values by factors between 3 and 100, in the range shown; DELTA-BLAST and PSI-BLAST by factors between 3 and 500. The search methods return similar numbers of false positives using nominal *E*-value cutoffs of 0.3 for BLASTP, 0.05 for CS-BLAST, 0.015 for PSI-BLAST, and 0.01 for DELTA-BLAST. We use these cutoffs in all comparisons of true positive results presented below.

### Search sensitivity comparison

**Figure 8:**
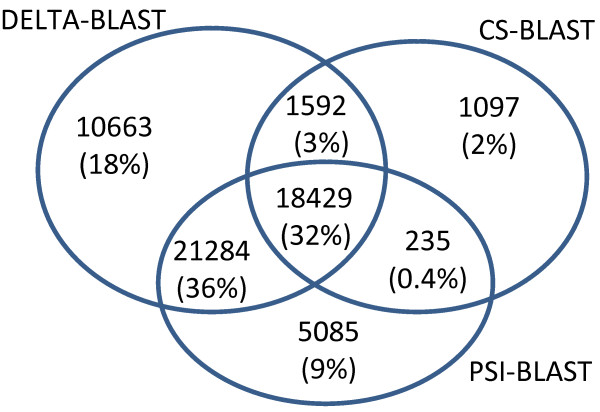
** True positives for DELTA-BLAST, PSI-BLAST, and CS-BLAST.** The Venn diagram shows the number of true positive results with nominal *E*-values below 0.01 for DELTA-BLAST, 0.015 for PSI-BLAST and 0.05 for CS-BLAST. The numbers in parentheses give percentages with respect to the total number of true positives found by all methods. Percentages do not sum precisely to 100% due to rounding.

We recorded the true positive (TP) results with nominal *E*-values below the method-specific thresholds (see above) returned by DELTA-BLAST, PSI-BLAST, and CS-BLAST, and summarize the results in Venn diagrams. Figure 8 shows the number of all TPs found by at least one of the three methods; about 32% of these are found by all methods. 18% are found only by DELTA-BLAST, and DELTA-BLAST finds 89% of the TPs. DELTA-BLAST and PSI-BLAST together find 98% of all TPs. CS-BLAST finds 37% of all TPs, and about 2% of TPs are found only by CS-BLAST. Excluding those TPs found by all methods, there is a relatively small overlap between PSI-BLAST and CS-BLAST (0.4%). An order of magnitude greater overlap arises between DELTA-BLAST and CS-BLAST (3%), and there is a large overlap between DELTA-BLAST and PSI-BLAST (36%).

**Figure 9:**
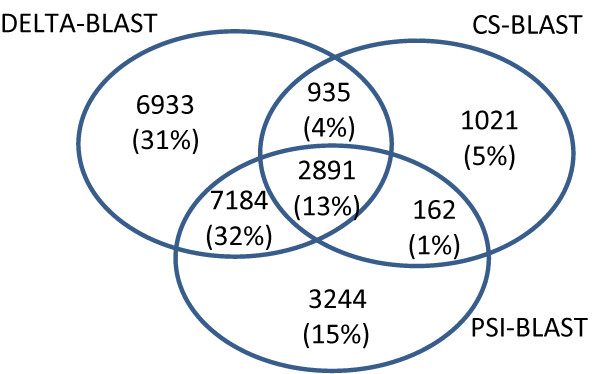
** True positives with query and subject sequences from different SCOP families.** The Venn diagram shows the number of true positive results with nominal *E*-values below 0.01 for DELTA-BLAST, 0.015 for PSI-BLAST and 0.05 for CS-BLAST, in which query and subject belong to different SCOP families.

Figure 9 presents the number of TPs involving distant sequences, *i.e.* those assigned to different SCOP families (see **Methods**). The fraction of TPs found by all methods is cut by more than half, to 13%, while the share of hits found by only a single method increases to 31% for DELTA-BLAST, 15% for PSI-BLAST, and 5% for CS-BLAST.

**Figure 10:**
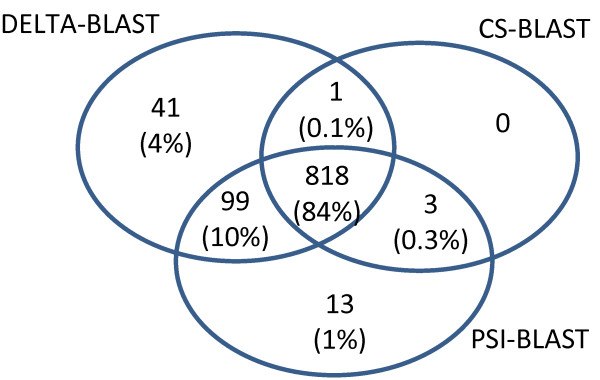
** Number of SCOP superfamilies yielding at least one true positive alignment.** The Venn diagram shows the number of SCOP superfamilies yielding at least one true positive result with nominal *E*-value below 0.01 for DELTA-BLAST, 0.015 for PSI-BLAST and 0.05 for CS-BLAST. Both query and subject sequence must come from the same superfamily.

The sensitivity of the search methods across SCOP superfamilies is summarized in Figure 10, which reports the number of superfamilies yielding at least one TP. All methods find at least one match in 84% of these superfamilies. There are 41 superfamilies with TPs found only by DELTA-BLAST, 13 only by PSI-BLAST, and none only by CS-BLAST.

### CDD annotation of true positive results

In this section, we compare the ability of the search methods to discover distant or novel homologies, using CDD as a repository of known homologies that are relatively easily modeled with sequence-based methods. We computed for each method the fraction of TPs with *E*-value up to each method-specific threshold, with query and subject sequences matching significantly to the same CD, the same CDD superfamily [26], different CDD superfamilies, or with at least one sequence matching no CD (see **Methods**). Table 4 presents the percentages of DELTA-BLAST, PSI-BLAST, BLASTP, and CS-BLAST TPs that fall into each of the above groups. The last row of Table 4 shows the fraction of all benchmark TPs (see **Methods**) that fall into each group.

**Table 4:** CDD annotations of true positive search results

		**Number and (%) of TPs for which (query, subject)**			
**Method**	**Number of TPs**	**Both match**	**Both match **	**Both match**	**Either matches**
		**same CD**	**same CDD**	**only different**	**no CD**
			**superfamily**	**superfamilies**	
DELTA-BLAST	51,968	48,612 (93.5)	49,001 (94.3)	2,521 (4.9)	446 (0.9)
PSI-BLAST	45,033	40,194 (89.3)	40,989 (91.0)	3,110 (6.9)	934 (2.1)
BLASTP	16,083	15,769 (98.1)	15,475 (96.2)	437 (2.7)	171 (1.1)
CS-BLAST	21,353	19,791 (92.7)	19,919 (93.3)	1,263 (5.9)	171 (0.8)
Benchmark TPs	196,490	60,663 (30.9)	95,073 (48.4)	88,994 (45.3)	12,423 (6.3)

The columns present: alignment program, number of true positives found, number and percentage of true positives for which a query and a subject both match the same CD, same CDD superfamily, only different CDD superfamilies, and for which either query or subject matches no CD. In the last row, the same data are given for all benchmark true positives.

The first two groups represent TPs for which the homologous relationship exists in CDD. They express the bias of the search methods towards known homologies. The third group represents TPs with remote homologs whose relationship is not modeled in CDD, and the last group represents TPs with at least one sequence not annotated in CDD. The last two groups assess the ability of a search method to detect remote and novel homologs.

The benchmark TPs elucidate the relationship between SCOP and CDD annotations and serve as a reference for comparison of TPs yielded by the search methods. About 31% of these pairs match the same CD, and 48% the same CDD superfamily. About 45% match different CDD superfamilies and 6% include at least one sequence matching no CDs. A divergence from this baseline indicates a bias of the search method towards homologs in one of the above groups.

Among the TPs returned by all methods a larger percentage match the same CD (at least 89%) or CDD superfamily (at least 91%) than is the case among all benchmark TPs. PSI-BLAST yields the smallest such percentage and BLASTP the largest. DELTA-BLAST yields a slightly larger percentage of TPs in these two groups than does CS-BLAST. The homologies for about 98% of BLASTP TPs are modeled by CDD.

All methods also yield a smaller percentage of TPs with sequences matching only different CDD superfamilies (at most 7%) than is the case among the benchmark TPs. PSI-BLAST yields the largest fraction of TPs falling into this group and BLASTP the smallest. The trend is similar for TPs with no CD match for at least one sequence. PSI-BLAST yields the largest percentage of TPs in this group and CS-BLAST the smallest. DELTA-BLAST yields only a slightly larger percentage of TPs in this group than CS-BLAST. In general, Table 4 demonstrates common biases among all the search methods towards TPs represented in CDD.

## Discussion

DELTA-BLAST outperforms BLASTP and CS-BLAST in homology detection by the measures both of ROC_*n*_ score and number of TPs found. In iterated searches, DELTA-BLAST also yields the best results. For individual searches, DELTA-BLAST provides high ROC_5_ scores for the largest number of test queries.

DELTA-BLAST outperforms other programs for all SCOP protein classes except for small proteins. The small protein sequences are often too short for RPS-BLAST to produce an alignment with statistically significant *E*-value, so few CDs are used in PSSM construction. It is surprising that CS-BLAST is outperformed by BLASTP, and CSI-BLAST by PSI-BLAST, for this protein class. One might expect that the application of short profiles in CS-BLAST would work well with small proteins.

Venn diagrams show that DELTA-BLAST is more sensitive at detecting both relatively similar and more distantly related sequences, *i.e.* those that belong to different SCOP families. DELTA-BLAST also detects homologous relationships in a larger number of SCOP superfamilies than do the other search programs.

The small intersection between the sets of TPs found by PSI-BLAST and CS-BLAST might be a result of their different approaches to constructing PSSMs. PSI-BLAST relies on homologous alignments, and CS-BLAST relies on short profiles, weighted by similarity to the query. DELTA-BLAST and PSI-BLAST use similar approaches, and the intersection between their sets of TPs is consistently the largest. The intersection between the sets of TPs found by DELTA-BLAST and CS-BLAST is smaller than for those of PSI-BLAST and DELTA-BLAST, but larger than for those of PSI-BLAST and CS-BLAST. DELTA-BLAST takes advantage of both homologous alignments and short conserved blocks since it uses ‘pre-constructed’ domain profiles.

DELTA-BLAST produces better quality alignments than do BLASTP and CS-BLAST. The differences are especially large for low sequence similarity (0–20% identity). In the range of 0 to 5% sequence identity, DELTA-BLAST’s alignments have average sensitivity twice as large as that of BLASTP and of CS-BLAST and precision greater by at least 0.05. Alignments to homologous conserved domains allow DELTA-BLAST to match a query sequence to existing PSSMs, and select appropriate domain models, even if the target protein family exhibits a low degree of residue conservation.

CDD annotations of true positives indicate that all search methods are biased towards homologies modeled in CDD. This bias is the strongest in BLASTP and DELTA-BLAST, and slightly smaller in CS-BLAST and PSI-BLAST. DELTA-BLAST, PSI-BLAST, and CS-BLAST rely on information from alignments of similar sequences from the NR database, which undergo additional processing in the construction of CDD and of the context profile library [24]. Therefore, their similar behavior is not surprising. BLASTP aligns closely related sequences, most of which match CDs.

Most benchmark sequences can be annotated with CDs, but only about half of homologous relationships are captured in CDD. Each of the search methods studied detects only a small fraction of the benchmark homologous pairs not modeled in CDD.

DELTA-BLAST finds more than twice as many TPs overall as does CS-BLAST. Accordingly, the number of TPs in which the query and subject match different CDD superfamilies is also larger for DELTA-BLAST. PSI-BLAST yields the largest fraction of TPs with the query and subject matching different CDD superfamilies and with no CDD annotation for the query or subject.

It is surprising that multiple iterations of DELTA-BLAST perform worse than does a single one. The reasons for this decline in performance include:

1) Saturation of search results: The number of significant alignments generated by the first DELTA-BLAST iteration often exceeds PSI-BLAST’s limit (5000) for inclusion in PSSM computation. This saturation may result in biased PSSMs, and a decline in performance in subsequent iterations.

2) Too much diversity in protein families: It is important to strike a balance between diversity and information in a search model seed alignment [35-38]. For large and diverse protein families, multiple PSSMs targeted to different subfamilies may be better for finding homologs than a single PSSM that tries to model the whole family. The SCOP superfamily c.37.1 (P-loop NTPases) is an example. A single iteration of DELTA-BLAST detects a large portion of this superfamily, due to CDs that model several of its families. After DELTA-BLAST’s second iteration, the MSA it produces is too diverse, causing the resulting PSSM to lose sensitivity.

DELTA-BLAST owes its superior performance to CDD. PSSMs are created from MSAs and constructing an appropriate MSA is critical for any profile-sequence-based search. DELTA-BLAST uses already prepared MSAs stored in CDD for the purpose of annotating protein sequences with conserved domains.

DELTA-BLAST performance, whether it is search sensitivity or quality of alignment, strongly depends on the quality and comprehensiveness of the CDD collection. Large numbers of CDs are manually curated to improve their MSAs as well as their sensitivity and specificity as search models. CDD also imports MSAs from other projects, which ensures a comprehensive database. CDD is an actively maintained resource that includes domain models derived using diverse methods, including ones based on structure and on sequence clustering. Between the February 2009 and August 2011 releases, the number of CDD domain models increased by 48%, with 86% growth in the NCBI-curated CDs, and the number of superfamilies increased by 27%. Currently, 78% of sequences in the NR database match some CDD domain model. We expect that DELTA-BLAST’s sensitivity and specificity will improve further with the growth of CDD, as new models are added and old ones improved.

Additionally, because CDD search is more sensitive than sequence search, DELTA-BLAST achieves better performance at finding appropriate models to construct a PSSM. Furthermore, manually curated MSAs are less likely to be corrupted by false positive matches as can be the case for a PSI-BLAST PSSM built on the fly. Many query sequences match more than one CD, allowing DELTA-BLAST to build a composite PSSM that may be more effective than the PSSMs associated with individual matching CDs. For sequences that do not match to any CDs, DELTA-BLAST performs a BLASTP search that can be iterated with PSI-BLAST.

## Conclusions

We have described DELTA-BLAST, a new tool for detecting distant homologs in a protein database search. The results of our experiments show that DELTA-BLAST detects more homologs and provides better quality alignments than do other programs analyzed in this paper.

DELTA-BLAST’s strategy is distinct from those of CS-BLAST and PSI-BLAST in a number of ways. It uses long, putatively homologous alignments with CDs to build its PSSMs, whereas CS-BLAST uses short (13-residue-wide), not necessarily homologous matches with context library profiles. PSI-BLAST performs a BLASTP search to produce alignments and build a PSSM without requiring a specialized or preprocessed resource, although it needs more time for this task. CDD requires more effort to maintain than does the CS-BLAST library of context profiles. However, CDD is an actively maintained resource that is already heavily used for protein annotation at NCBI.

We are exploring ways to improve DELTA-BLAST’s performance, *e.g.* by developing better methods for weighting coincident hits to several CDs, and by using more information stored in CDD, such as domain hierarchies and specific hit scores (see [26] for details). Since selecting appropriate CDs is at the heart of DELTA-BLAST’s performance, we are also exploring improvements to RPS-BLAST, and the use of different CDD search tools. Our initial experiments suggest that GLOBAL [29] works very well for several SCOP superfamilies that are problematic for RPS-BLAST. We also plan to address the performance decline in iterated DELTA-BLAST by using approaches that maintain the information content in the PSSMs constructed.

## Methods

DELTA-BLAST constructs a PSSM by combining profile information from conserved domains related to a query sequence, and then searches a sequence database with this PSSM. The following subsections provide a more detailed description of the algorithm and of the databases and experiments used to assess it.

### Query and database sequences

We evaluated the performance of DELTA-BLAST using the ASTRAL 40 subset [30] of release 1.75 of the Structural Classification of Proteins (SCOP) [31] database. To create a query set, we sorted the SCOP domains in lexicographic order and divided them into training (odd numbered sequences) and test (even numbered sequences) query sets. We removed from these sets any sequences that were the sole member of their superfamily in ASTRAL 40. We used the training set to optimize parameters, and the test set to evaluate DELTA-BLAST performance. The “benchmark” database, against which the queries were searched, consisted of all ASTRAL 40 sequences.

For a given query sequence, we ignored its self hit but counted as a homolog (true positive), any benchmark sequence belonging to the same SCOP superfamily, and as a non-homolog (false positive) any benchmark sequence belonging to a different SCOP fold. We did not classify as either a true or false positive any sequence belonging to the same fold but a different superfamily, because it is difficult to establish whether or not such a sequence is homologous.

There were 4853 and 4852 queries in the training and test sets, respectively, and the benchmark database contained 10,569 sequences. The training set yielded 195,944 homologous pairs, and the test set 196,490.

### Conserved domains

In this work, CDD is both a domain annotation resource and a collection of protein family profiles used for building sequence search models. Each conserved domain (CD) within CDD consists of a multiple sequence alignment (MSA). Each column of these MSAs is characterized by weighted observed frequencies for the various amino acids as well as by an effective number of independent observations [23,34,39].

To avoid constructing PSSMs that are narrowly focused, we consider only CDs that are sufficiently diverse. Specifically, we exclude any CD for which the maximum number of independent observations, measured across all columns, is less than 6. We found that for DELTA-BLAST this threshold yielded the best homology detection performance on our benchmark set.

After employing RPS-BLAST to compare a query sequence to CDD, DELTA-BLAST uses a matching CD for PSSM construction only if its reported *E*-value falls below a specified threshold. Empirically, the threshold 0.05 yielded the best balance between search sensitivity and the potential for the resulting PSSMs to be corrupted [34] when tested on our training set.

### Multiple alignment of conserved domains

To construct an MSA from CDs, we first collect all CD segments that have been aligned to the query with *E*-value below a user-specified threshold. Analogously to PSI-BLAST, we use the query as a template for collapsing multiple pairwise alignments into a single MSA and then constructing a corresponding PSSM. CD columns that align to gaps inserted into the query are ignored. If the same CD aligns more than once to a given column of the query sequence, only the alignment with the lowest *E*-value is retained for that column.

**Figure 11:**
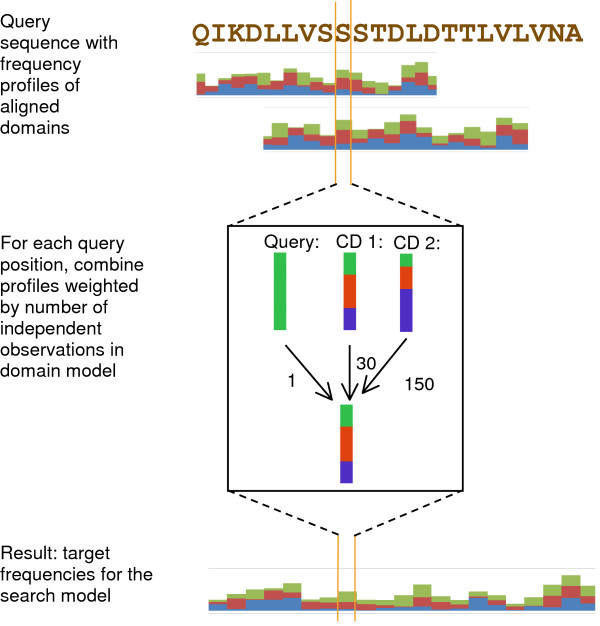
**Overview of computing the target frequencies for a PSSM position.** Amino acid frequency profiles of conserved domains aligned to the query are added after weighting by the number of independent observations in domain models (shown as numbers next to the arrows). The query sequence is included, with one observation, in all positions where the query residue was not observed in any aligned domain.

After alignment to any CDs, the query sequence forms part of a multiple alignment, represented with a single residue count in each column. To avoid over-representing the query sequence in the constructed PSSM, we refrain from tallying this count when the query’s residue is already represented in an aligned CD. This preserves the effectiveness of CDs that model large protein families, but ensures that all residues in the query sequence enter into the construction of the resulting PSSM. The estimation of residue frequencies in a column is depicted in Figure 11.

### The derivation of target frequencies and scores for PSSMs

The PSSM scores for any column all take the form (ln *R*_*i*_*)/λ*, where *R*_*i*_ is the ratio of target to background frequency for residue *i*, and λ is a scaling factor [23].

We follow PSI-BLAST’s procedure for estimating frequency ratios [34,39], which is based on the “data-dependent pseudocount” method [40] for addressing the issues of small sample size and prior knowledge of amino acid relationships.

Fong and Marchler-Bauer [41] note that alignment *E*-value does not provide good criterion for selecting among or weighting matching homologous domains. Therefore, we weight aligned CD columns not as a function of their match score, but rather proportionately to their effective number of independent observations. To obtain statistical parameters [42] for the resulting PSSM used with gapped scores, we use the scaling procedure presented in [23].

### Assessment

We describe here our protocol for comparing the performance of DELTA-BLAST, by various criteria, to those of BLASTP, PSI-BLAST version 2.2.25, and Context-Specific BLAST (CS-BLAST) version 2.1.2. The CDD database used by DELTA-BLAST is a subset of CDD version 2.27.

#### Retrieval accuracy

After comparing a query set to the benchmark database, we pooled all search results, ordering them by nominal *E*-value. We measured retrieval accuracy on the resulting list using the ROC_*n*_ score, the normalized area under the ROC curve up to *n* false positives [33]. The ROC_*n*_ score has value between zero and one, with larger scores denoting better performance; we calculate standard errors as described in [34]. Then we divided the pooled results into subsets according to the SCOP protein class of the query and computed ROC_*n*_ score for each subset, with *n* equal to the number of subset queries.

We also computed ROC_5_ scores from the search results for each query and plotted the percentage of all test queries that yield results with ROC_5_ score larger than a given value vs. the value. To ensure that we could compute ROC scores for up to five false positives, we added five fake false positive results at the end of each results list.

To construct effective PSSMs, PSI-BLAST must search a comprehensive sequence database. Therefore, to evaluate PSI-BLAST, we used it on all initial iterations to search a version of the NCBI’s Non-Redundant (NR) database frozen on August 16, 2011, and to search the benchmark database only on the final iteration.

We examined as well the performance of PSI-BLAST when initialized with a CS-BLAST or a DELTA-BLAST generated PSSM. We refer to these methods as Context-Specific Iterated BLAST (CSI-BLAST) [24], and iterated DELTA-BLAST. *i* iterations of CSI-BLAST or iterated DELTA-BLAST refers to *i – 1* iterations of PSI-BLAST started from a CS-BLAST or DELTA-BLAST computed PSSM.

For PSI-BLAST, CSI-BLAST, and iterated DELTA-BLAST we set to 5000 the maximum number of PSI-BLAST search results from the previous iteration used for PSSM construction. We selected this number for consistency, because it is hard-coded into the CSI-BLAST program [24].

#### Alignment quality

We assessed alignment quality using the superfamily subset of the SABmark set [32]. SABmark provides the reference alignments for sequences that correspond to SCOP domains. In this experiment, we used only alignments with sequence identity in the reference alignment below 30%. Additionally, we removed alignments that contained at least one sequence present in the training set used to tune DELTA-BLAST parameters, and alignments with fewer than five aligned pairs of residues. The resulting set contained 10,006 alignments between 2,379 sequences.

We generated sequence alignments for each pair of sequences with DELTA-BLAST, BLASTP, and CS-BLAST. The best-scoring alignment was assessed for each pair. The quality of sequence alignments was measured by alignment sensitivity defined as |N∩S|/|S|, and precision defined as |N∩S|/|N|, where *N* is the set of residue pairs in the sequence alignment, and *S* is the set of residue pairs in the reference alignment. These alignment quality measures are identical to the *f*_*D*_ and *f*_*M*_ measures used in [43].

We grouped the alignments into bins by sequence identity in the reference alignment, and calculated average sensitivity and precision for each bin.

#### Accuracy of nominal *E*-values

For each search method, we graphed against *x* the mean number of false positive hits with nominal *E*-value ≤ *x*. Because the *E*-values reported by different methods are not equally accurate, we identified nominal *E*-value thresholds at which the various methods return similar numbers of false positives. These cutoffs are used in the experiments described below.

#### Search sensitivity comparison

We compared DELTA-BLAST, PSI-BLAST, and CS-BLAST sensitivity by the number of true positive hits found with *E*-value below the respective method-specific thresholds (see above). We also compared the number of such hits with query and subject belonging to different SCOP families, and the number of superfamilies yielding at least one true positive hit. We ran PSI-BLAST in the same manner as described in the ***Retrieval accuracy*** subsection, but report results only for five iterations.

#### CDD annotation of true positive results

We annotated the test set and database sequences by using RPS-BLAST to compare them to CDD version 2.30. An *E*-value ≤ 0.01 yielded an association with a CD. For DELTA-BLAST, PSI-BLAST, CS-BLAST, and BLASTP, we examined the CDD associations of true positive hits with *E*-values below the respective method-specific thresholds. For each method, we recorded the fraction of the true positives returned with the query and subject annotated with the same CD, same CDD superfamily, different CDD superfamilies, and with either query or subject matching no CD. We computed the same percentages for all homologous pairs in the benchmark set. An association of both query and benchmark sequence with at least one common CD yielded assignment to the first of these groups, while an association of both with at least one common CDD superfamily yielded assignment to the second. Because not all CDs belong to CDD superfamilies, the frequencies for the same and different superfamilies may not sum to one.

## Competing interests

The authors declare that they have no competing interests.

## Authors’ contributions

DJL conceived the project. GMB, TLM, and AAS designed the algorithm. AAS, DJL, RA, and SFA suggested experiments. GMB implemented the algorithm and conducted experiments. GMB and TLM prepared the first draft. AAS, SFA, and RA gave substantial contributions in drafting and revising the paper. All authors read and approve the final manuscript.

## **Reviewers’ comments**

*Reviewer’s report 1*

Arcady Mushegian, Stowers Institute for Medical Research, Kansas City, MO, USA

The manuscript by Boratyn et al. describes a new addition to the BLAST family of programs. The main idea of DELTA-BLAST is to start sequence comparisons with matching the query sequence not to the individual homologous sequences in the peptide database (as PSI-BLAST does), but to models of conserved sequence domains in the domain database (in this case, the NCBI CDD database), and to build a probabilistic family model (PSSM) from the alignment of the query to highly-scoring domain model. The PSSM is then submitted to a round of sequence database search, same as in PSI-BLAST. The majority of the manuscript is devoted to benchmarking the performance of DELTA-BLAST against PSI-BLAST and CS-BLAST (an approach, developed by J. Soding’s group, similar to DELTA-BLAST but relying on the library of patterns that is shorter and may be less well curated than CDD).

The authors have outstanding track record in improving methods of sequence database search, and I am sure that DELTA-BLAST, too, will find its uses. I feel, however, that the paper’s focus on benchmarking leaves several more substantive questions not very well answered. My concerns are along three lines, i.e., what is the scientific problem that DELTA-BLAST is aimed at solving; why does it work; and how it is integrated with the suite of other BLAST programs.

Authors’ response

We provided answers to the above concerns below, answering Reviewer’s more detailed questions.

One goal of the effort seems to be finding more homologs of a given query than in PSI-BLAST searches. Is that indeed so? In the later sections of the Results and in Discussion, we also see notes on better alignment quality in DELTA-BLAST, and on the ability of DELTA-BLAST to classify the query sequence (“classify” is not defined – does this mean “detect a CD that is the closest match to the query”? Obviously, that would be not the same as to say that the query is an in group of that CD family – it may be an out group). Which of these goals are primary, and which are more of auxiliary benefits?

Authors’ response

*We added the following explanatory text as the sixth paragraph of****Background****: “Our primary goals for DELTA-BLAST are to make use of a PSSM in the search (as in PSI-BLAST) to find more homologs, but to avoid the time spent in the initial BLASTP search. DELTA-BLAST also allows us to explore whether it is better to use longer homologous alignments to quickly construct a PSSM than the short profiles of Biegert and Söding*[24]*. In future work, it may serve as a platform to experiment with different methods for quickly finding initial matches to a query that can then be used to construct a PSSM.”*

*The quality of alignments produced by DELTA-BLAST is an auxiliary benefit. Classification of protein sequences was never our goal. We used the term ‘classify’ to describe the first step in DELTA-BLAST, i.e. finding CDs that model a query sequence. We replaced the two occurrences of the word ’classify’ in ***Background and Discussion*** to avoid this confusion.*

A related concern is: why aligning a query to a PSSM of of matching conserved domains is a better first step in the search strategy (better in the most important sense, i.e., ensuring better sensitivity) than building a PSSM from matching sequences, as PSI-BLAST does? Sometimes the first BLAST search of sequence databases produces no above-the-threshold similarities, and therefore nothing to build a PSSM with, whereas RPS-BLAST gives a significant match to one or more CDs, enabling one to construct a PSSM; I understand that in these cases, DELTA-BLAST would be more sensitive than PSI-BLAST. But many other times, the CD to which query actually belongs has not been described yet and is not in CDD, and yet PSI-BLAST is finding some homologs in the database, allowing a PSSM construction and iterative search. In these cases, PSI-BLAST is sensitive and DELTA-BLAST is moot.

Authors’ response

*We added the following two paragraphs at the end of****Discussion****: “PSSMs are created from MSAs and constructing an appropriate MSA is critical for any profile-sequence-based search. DELTA-BLAST uses already prepared MSAs stored in CDD for the purpose of annotating protein sequences with conserved domains. DELTA-BLAST performance, whether it is search sensitivity or quality of alignment, strongly depends on the quality and comprehensiveness of the CDD collection. Large numbers of CDs are manually curated to improve MSAs as well as their sensitivity and specificity as search models. CDD also imports MSAs from other projects, which ensures a comprehensive database.”*

“Additionally, because CDD search is more sensitive than sequence search, DELTA-BLAST achieves better performance at finding appropriate models to construct a PSSM. Furthermore, manually curated MSAs are less likely to be corrupted by false positive matches as can be the case for a PSI-BLAST PSSM built on the fly. Many query sequences match more than one CD, allowing DELTA-BLAST to build a composite PSSM that may be more effective than the PSSMs associated with individual matching CDs. For sequences that do not match to any CDs, DELTA-BLAST performs a BLASTP search that can be iterated with PSI-BLAST.”

For queries that do not match any CDs, our initial small scale experiments suggested that it is beneficial in some cases to construct a PSSM using possibly non-homologous segments of CDs. This can be done by increasing DELTA-BLAST’s domain inclusion E-value threshold (a user controlled parameter). This requires more thorough research that we plan to do in the future. Furthermore, if a query does not match any CDs, DELTA-BLAST defaults to PSI-BLAST.

Was the testing described in the study done on the sequences that mostly followed the former scenario? If so, why? Is a random subset of sequences from protein database dominated by sequences that are already assigned to CD?

Authors’ response

Yes, the testing was done with a set with majority of sequences having an assigned CD. Large-scale experiments that involve different types of proteins require a benchmark set with known homologies. Unfortunately, such a set will often include known proteins and many known proteins are already assigned to a CD. Currently, about 78% of sequences in the NR database match at least one CD with the E-value below 0.01.

The performance was tested on a relatively small set of queries and relatively small database, and it is possible that both are indeed strongly enriched by sequences with known domain composition. Have there been any tests that mimic other common use cases, e.g., the set of queries is a complete list of proteins encoded by newly sequenced genomes, or the database is NR or all proteins encoded by genomes in GenBank Genome division? Would the gain in sensitivity by DELTA-BLAST be the same?

Authors’ response

*We performed the experiments presented in the manuscript on a gold standard benchmark set with known homologies, so that search accuracy could be compared with results presented in other publications. To mimic other common uses we looked at the second iteration PSI-BLAST searches submitted through the NCBI BLAST web page between February 6 and February 13, 2012. Out of 1064 unique sequences submitted during this time 73% matched at least one CD. We also selected four recently sequenced genomes from diverse taxonomic nodes: Archaea (*http://www.ncbi.nlm.nih.gov/genome/11226*), Bacteria (*http://www.ncbi.nlm.nih.gov/genome/12533*), Eukaryota (*http://www.ncbi.nlm.nih.gov/genome/11437*), and Virus (*http://www.ncbi.nlm.nih.gov/genome/12485*), and computed the fraction of protein sequences that match at least one CD with E-value below 0.05 (default threshold for DELTA-BLAST). 67% of the 2835 protein sequences in the archaeal genome match a CD. For the bacterial genome, 78% of the 3881 sequences align to a CD. 85% out of the 4434 sequences in the eukaryotic genome and 36% of the 105 sequences in the virus genome match at least one CD. We expect that DELTA-BLAST would provide improved sensitivity for the above sets of sequences, although the gain would probably be smaller than for our benchmark set.*

Finally, it would be helpful to describe better the software offering — is it integrated with other BLAST programs in any way? Most immediately, if there are no matching CD, will the program default to PSI-BLAST automatically?

Authors’ response

*We added the following explanatory text at the end of****Background****: “DELTA-BLAST is fully integrated with the NCBI BLAST website and the stand-alone BLAST+ package. It is available from the ‘Protein BLAST’ link at the NCBI BLAST website (*http://blast.ncbi.nlm.nih.gov*). A DELTA-BLAST search on the website can be followed up by PSI-BLAST iterations or the results can be processed further by the distance tree or multiple alignment tools. A new program named deltablast will be part of the command-line BLAST+ package starting with the 2.2.26+ release. Source code and applications for popular platforms are available at*ftp://ftp.ncbi.nlm.nih.gov/blast/executables/blast+/LATEST/*.”*

*We also added the last sentence in****Discussion****: “For sequences that do not match to any CDs, DELTA-BLAST performs a BLASTP search that can be iterated with PSI-BLAST.”*

Quality of written English: Acceptable

*Reviewer’s report 2*

Nick V Grishin, University of Texas Southwestern Medical Center, Dallas, TX, USA

The main product of this work is a piece of software from the BLAST family that without iterations achieves and possibly surpasses iterated PSI-BLAST performance on ASTRAL superfamilies, thus allowing faster and likely more accurate sequence database searches. Just this fact alone is enough to raise interest of researchers. The suggested innovation is that prior to sequence database search, the new software does CDD search to find homologous families, and uses their pre-computed and curated alignments to seed sequence database search.

On the conceptual level, the authors argue that seeding the search with pre-compiled alignments of homologous families is advantageous to seeding the search with short, possibly non-homologous segments similar to the query sequence. This logical statement is firmly supported by comparing their new program, DELTA-BLAST, with CS-BLAST. However, it might be interesting to study whether there is any advantage in combining the two techniques, and whether adding short segment profiles might help searches when homologous profiles in CDD are either very thin or not found.

Authors’ response

We thank the Reviewer for this suggestion. Our small scale experiments suggested that using short CD segments possibly non-homologous to a query may improve DELTA-BLAST sensitivity when there are no strong CD matches. We plan to research this idea further.

My main concern, as always, is with validation. I fully agree with the authors that validation presented is enough to derive main conclusions sought. 1) DELTA-BLAST is not worse, and might be even better than PSI-BLAST in some occasions. Indeed, why would it be worse? It is the same thing, but seeded with more accurate curated CDD alignments. 2) DELTA-BLAST outperforms CS-BLAST, and how could it not? Homologous profiles are expected to be more powerful. However, beyond these conclusions it might not be possible to understand behavior of the three programs better, for the following reasons:

1. ASTRAL superfamily dataset is not ideal. According to SCOP, proteins placed in the same superfamily are homologous. However, it is not stated by SCOP authors that proteins from different superfamilies and even folds are not homologous. Indeed there are many homologous proteins in different superfamilies and folds, e.g. many proteins in a/b class (Rossmann-like folds) are most likely homologous regardless of the fold they are placed into, and their detection by sequence search software with an alignment that matches structure-based alignment should not be counted as “false positive”. Moreover, not performing evaluation on a very rich dataset of pairs within the same fold, but in different superfamilies, the authors neglect the most interesting “gray” area of sequence search their sensitive approach is targeted for, and skew performance statistics. I.e. the majority of protein pairs are thrown away from this evaluation.

Obviously, it is difficult to deal with these pairs, because some of them are homologous, while others are not. However, approaches have been proposed in the literature to deal with this problem.

Authors’ response

We agree with the critique and we plan to perform more experiments in the future. We used a gold standard data set used in other publications, so that results can be compared.

2. ROC curve on all data pulled together might not be fully informative. It might be skewed towards families with longer sequences and thicker profiles that attain lower E-values. Thus the ROC-region shown might be dominated by Rossmann folds and P-loop proteins. It might be worth comparing how different programs rank hits for each query, e.g. by checking ROCx plots – fraction of queries with ROCx score above a given value vs. the value. ROCx score is the ratio of the area under the ROC curve up to x-th false positive to the area under ideal ROC curve. x is usually small, e.g. around 5.

Authors’ response

*We included the ROC*_*5*_*plot suggested by the Reviewer in Figure*4*along with appropriate text in****Results****(two last paragraphs in subsection****Homology detection****),****Discussion****(the third sentence), and****Methods****(the second paragraph in****Retrieval accuracy****).*

3. Different protein types may show different behavior. Is there a dominant fold type contributing to the ROC curve? Could it be Rossmann-like and P-loop proteins? What is the performance in different protein classes?

Authors’ response

*We computed the fraction of true positive pairs for each SCOP fold as a part of all true positive pairs in the benchmark set. P-loop containing nucleoside triphosphate hydrolases (c.37) has the largest share of true positive pairs: 15%. The share for DNA/RNA-binding 3-helical bundle (a.4) is 11%, NAD(P)-binding Rossmann-fold domains (c.2) 9%, and Immunoglobulin-like beta-sandwich (b.1) is 9%. Other folds have much smaller share and the distribution of the number of true positive pairs per fold has a long tail with many folds with relatively small number of pairs. As suggested by the Reviewer, we included in the manuscript Table*3*which shows ROC*_*n*_*scores computed for SCOP classes along with appropriate text in****Results****(the fourth paragraph in subsection****Homology detection****),****Discussion****(the second paragraph), and****Methods****(the last sentence of the first paragraph in****Retrieval accuracy****).*

I am not suggesting to address all these concerns in the present study, however, these points might be worth considering in future work.

As a minor problem, it might be instructive to the readers, especially biologists, to clarify what proteins hide behind the code name “superfamily c.37.1”. It is P-loop NTPases, a very special and interesting group.

Authors’ response

We added “(P-loop NTPases)” next to the single occurrence of c.37.1 in the manuscript text.

*Reviewer’s report 3*

Frank Eisenhaber, Bioinformatics Institute, Singapore

The authors propose another variant of the successful BLAST suite of programs for similarity searches among protein sequences. The weak point of PSI-BLAST was the automated simplified generation of multiple alignments and their sometimes non-satisfactory quality was one of the main reasons why the program did not find certain homologues. Not surprisingly, competitive development such as CS-BLAST attempted to improve the alignment construction by using specially created libraries. The idea to rely on theexisting large collection of manually curated alignments provided by CDD is a nice workaround and certainly worth pursuing.

The authors provide an exhaustive assessment of the accuracy/sensitivity of their tool and it looks quite convincing that the large alignment library indeed boosts the likelihood of finding homologues.

Quality of written English: Acceptable

Authors’ response

We thank the Reviewer for these comments.
